# Using Cost-Effectiveness Analysis to Address Health Equity Concerns

**DOI:** 10.1016/j.jval.2016.11.027

**Published:** 2017-02

**Authors:** Richard Cookson, Andrew J. Mirelman, Susan Griffin, Miqdad Asaria, Bryony Dawkins, Ole Frithjof Norheim, Stéphane Verguet, Anthony J. Culyer

**Affiliations:** 1 Centre for Health Economics, University of York, York, UK; 2 Academic Unit of Health Economics, Leeds Institute of Health Sciences, University of Leeds, Leeds, UK; 3 Department of Global Public Health and Primary Care, University of Bergen, Bergen, Norway; 4 Department of Global Health and Population, Harvard University, Harvard T.H. Chan School of Public Health, Boston, MA, USA

**Keywords:** cost-effectiveness analysis, delivery of health care, health equity, technology assessment

## Abstract

This articles serves as a guide to using cost-effectiveness analysis (CEA) to address health equity concerns. We first introduce the "equity impact plane," a tool for considering trade-offs between improving total health—the objective underpinning conventional CEA—and equity objectives, such as reducing social inequality in health or prioritizing the severely ill. Improving total health may clash with reducing social inequality in health, for example, when effective delivery of services to disadvantaged communities requires additional costs. Who gains and who loses from a cost-increasing health program depends on differences among people in terms of health risks, uptake, quality, adherence, capacity to benefit, and—crucially—who bears the opportunity costs of diverting scarce resources from other uses. We describe two main ways of using CEA to address health equity concerns: 1) equity impact analysis, which quantifies the distribution of costs and effects by equity-relevant variables, such as socioeconomic status, location, ethnicity, sex, and severity of illness; and 2) equity trade-off analysis, which quantifies trade-offs between improving total health and other equity objectives. One way to analyze equity trade-offs is to count the cost of fairer but less cost-effective options in terms of health forgone. Another method is to explore how much concern for equity is required to choose fairer but less cost-effective options using equity weights or parameters. We hope this article will help the health technology assessment community navigate the practical options now available for conducting equity-informative CEA that gives policymakers a better understanding of equity impacts and trade-offs.

## Introduction

Health equity has risen to prominence on policy agendas in the wake of the universal health coverage movement [1], [2], [3] and landmark international reports on inequality in health [4], [5] and health care [3], [6], [7]. The cost-effectiveness analysis (CEA) studies that are routinely used around the globe to inform priority setting in health care and public health, however, rarely provide information about who gains and who loses from health programs or about trade-offs between cost-effectiveness and equity in the distribution of health-related outcomes [8], [9], [10], [11], [12].

In recent years there have been a number of methodological advances in this area, which have been developed into practical tools, including extended cost-effectiveness analysis and distributional cost-effectiveness analysis [13], [14]. This article describes those tools and uses illustrations from high-, middle-, and low-income countries to demonstrate how they can be used to generate useful new information for decision makers about health equity impacts and trade-offs. In so doing, we part company from a venerable school of thought in public finance, according to which economic analyses of specific public expenditure programs and regulations should focus on potential Pareto efficiency in the sense of the Hicks-Kaldor compensation test and leave equity as a matter for income redistribution through the general taxation and social security system [15], [16].

Implicitly or explicitly, all CEA studies already incorporate social value judgments about equity—for example, in scoping and methodologic decisions about the relevant policy options and comparators, which costs and effects to measure, how to compare costs and effects of different kinds, how to aggregate costs and effects for different people and organizations, how to value future costs and effects, and so on [17]. These value judgments are rarely mentioned in applied CEA studies or health technology assessment (HTA) reports but are extensively discussed in textbooks, methods guidance documents, and other underpinning literature [18]. This article shows how to go beyond this standard approach of incorporating prespecified value judgments about equity within applied CEA studies, moving instead toward using CEA techniques to generate new information about the health equity implications of alternative policy options that facilitate deliberation among decision makers and stakeholders [8], [18]. Equity-informative economic evaluation is an input into decision-making processes, not an algorithm for determining decision outcomes [18]. The appropriate weight to give equity considerations in a particular decision is not a matter for analysts to resolve, but something for decision makers to consider in consultation with stakeholders.

We focus on two general categories of policy concern for health equity, which can both be used to address a wide range of more specific concerns: 1) reducing social inequalities in health and financial protection from ill-health; and 2) prioritizing the severely ill. Within the first category, our illustrative examples focus mainly on distributional impacts according to socioeconomic, ethnic, and sex groups, although the methods described are applicable to other differences in health-related outcomes that policymakers may consider unfair, including differences by geographical location, disability, mental illness, and other social variables.

First, we introduce two key concepts that underpin the economic approach to health equity analysis: 1) health equity trade-offs and 2) net equity impact. We then describe two approaches to conducting equity-informative CEA: 1) equity impact analysis, which quantifies the distribution of costs and effects by equity-relevant variables; and 2) equity trade-off analysis, which quantifies trade-offs between improving total health and other equity objectives.

## Health Equity Trade-offs

The policy objective underpinning conventional CEA can be thought of as a health equity objective: the quasi-utilitarian objective of maximizing total health in the general population [19], [20]. CEA compares the costs and effects of two or more mutually exclusive policy options [21]. To facilitate comparison between policies in different disease areas with diverse and distinct mortality and morbidity impacts, health effects are often measured using a composite summary index of health, such as the quality-adjusted life-year (QALY) or the disability-adjusted life-year (DALY). This allows the comparative effectiveness of programs to be assessed in terms of both individual and population-level health.

Population-level health gain is simply the unweighted sum total of all individual health gains, based on the standard value judgment that “a QALY is a QALY.” This also allows the calculation of an incremental cost per QALY gained, or per DALY averted, of one policy option compared with another. A cost-increasing policy option can be considered cost-effective if its cost per unit of health gain compares favorably with alternative ways of using resources. This recognition of opportunity costs—that resources used in the provision of a program would have generated value if used elsewhere—is fundamental to CEA.

Every benefit attributed to a program must be assessed relative to those displaced when resources are diverted from alternative activities. In a public health system with a fixed budget, the displaced activities will comprise alternative health programs that would have produced alternative health benefits. Cost-effectiveness can then be defined as a test of whether a program will improve total health. A cost-effective policy will have a positive net health impact because its health gains will outweigh the health losses that result from shifting expenditure away from other health programs. By contrast, a cost-ineffective policy will have a negative net health impact because the health losses that result from shifting expenditure away from other health programs will outweigh the health gains.

CEA can thus help decision makers to choose cost-effective investments that increase total health and avoid cost-ineffective investments that reduce total health. This interpretation of opportunity costs in terms of forgone health benefits is more problematic if there is no fixed health budget. Opportunity costs then may fall instead on household consumption (via increased taxes or insurance premiums) or on reductions in public expenditure on programs not primarily designed to improve health. Regardless, thinking about trade-offs between cost-effectiveness and health equity is useful even if the test of cost-effectiveness, or value for money, is not interpreted in terms of health maximization.

**Fig. 1:**
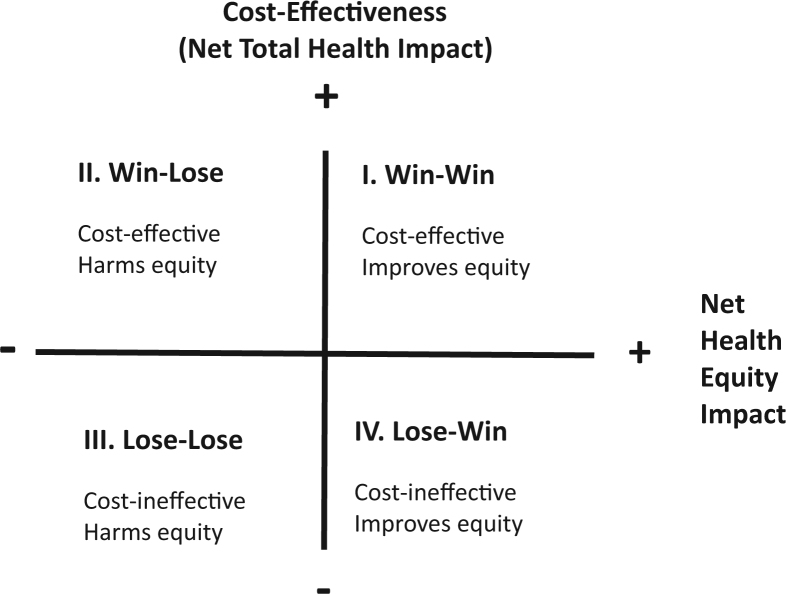
Health equity impact plane.

The health equity impact plane in Figure 1 is a simple way of thinking about the potential trade-offs between cost-effectiveness and an alternative health equity objective, such as reducing inequality in lifetime health or giving priority to those who are currently severely ill. The vertical axis shows the cost-effectiveness of a health program. As explained, it is often useful to think of cost-effectiveness as a measure of net total health impact: the total health benefits of the program minus the forgone health benefits that would have been obtained by spending the same money on other health programs.

The horizontal axis shows the net health equity impact of the program. This refers to the net impact on the alternative health equity objective, again after allowing for program opportunity costs as well as program benefits [21]. The net equity impact may be assessed informally by the decision maker in light of disaggregated information or by using formal health equity metrics that combine disaggregated information in a summary index [22], [23]. Different equity metrics can yield different conclusions, and the choice of metric requires justification based on explicit value judgments about a number of difficult conceptual questions, including equality of what? (e.g., outcome or opportunity), equality between whom? (e.g., all individuals or particular social groups), and equality indexed how? (e.g., absolute or relative indices) [24], [25]. In practice, the choice of metric will often reflect pragmatic considerations of data availability as well as value judgments—for example, because opportunity is hard to measure, health outcomes may sometimes be a useful proxy indicator for impacts on health opportunities [26].

In Figure 1, a policy that falls in quadrant I improves both total health and equity (“win-win”); in quadrant III, the policy harms both (“lose-lose”). In these two cases, the impacts on health maximization and health equity are in the same direction, so trade-offs are irrelevant. In contrast, in the other two quadrants, impacts on health maximization and equity are opposed and trade-offs become relevant. In quadrant II, the policy is good for total health but bad for equity (“win-lose”), and in quadrant IV, the policy is bad for total health but good for equity (“lose-win”). If all policies fell in quadrants I and III (“win-win” and “lose-lose”), there would be no need to analyze health equity impacts. The policy identified as cost-effective using standard CEA would always improve health equity, and a cost-ineffective policy would always harm health equity.

Many policies do indeed fall into quadrants I and III. In low- and middle-income countries, for example, investments in high-cost hospital treatments may fall into the “lose-lose” quadrant of being neither cost-effective nor likely to improve health equity insofar as they deliver small health gains per unit of cost and disproportionately benefit well-off groups [27]. In contrast, vaccination programs (e.g., rotavirus immunization [28]) and infectious disease control programs (e.g., tuberculosis [13]) may fall into the “win-win” quadrant of delivering large health gains per unit cost and reducing health inequity insofar as they disproportionately benefit socially disadvantaged groups.

In some cases, however, socially disadvantaged groups may gain less than advantaged groups from a decision to fund a particular medical technology as a result of factors such as unequal access to or quality of health care. As a case in point, access costs may be relatively high and health care coverage relatively low in remote, rural areas that lack well-resourced health facilities. In such cases, there may be trade-offs between cost-effectiveness and health equity. Policy makers may wish to consider redesigning delivery strategies to increase utilization and quality in disadvantaged communities, and may even wish to consider equity-oriented strategies that are less cost-effective than standard delivery strategies and lie in the “lose-win” quadrant.

Equity trade-offs can also arise in relation to preventive health interventions that seek to change behavior—including participation in screening and vaccination campaigns, as well as changes in smoking, diet, physical exercise, and other lifestyle behaviors—insofar as it may be more challenging and costly to change behavior in relatively disadvantaged communities, so preventive interventions may have more success in improving health in advantaged communities [29], [30], [31]. Such circumstances can give rise to preventive public health programs that lie in the “win-lose” quadrant (cost-effective but harmful to health equity), a phenomenon sometimes referred to as intervention-generated inequality [29].

This health equity impact plane provides a simple illustration for understanding when trade-offs are necessary. It can also be operationalized to present this information to decision makers, which has been done in two recent examples from the United Kingdom [32], [33].

## Net Equity Impacts

When assessing equity impacts, both the distribution of benefits and the distribution of opportunity costs are important. The forgone health benefits that could have been generated through the next-best alternative may be unequally distributed, and this distribution is required to estimate the net distributional health impact of a program [34]. The distribution of opportunity costs will depend crucially on how a program is funded. For example, if a program is funded by increasing general, progressive taxation, the absolute financial costs will be borne disproportionately by the rich, although the opportunity costs in terms of losses in health and well-being may be more equally distributed. In contrast, if a program is funded by reducing public expenditure on other health, education, or welfare services, the opportunity costs in terms of losses in health and well-being may be borne disproportionately by poorer individuals who rely more heavily on public services. The same applies to foreign aid funding that would otherwise be used to fund alternative programs that disproportionately benefit more socially disadvantaged people.

**Fig. 2:**
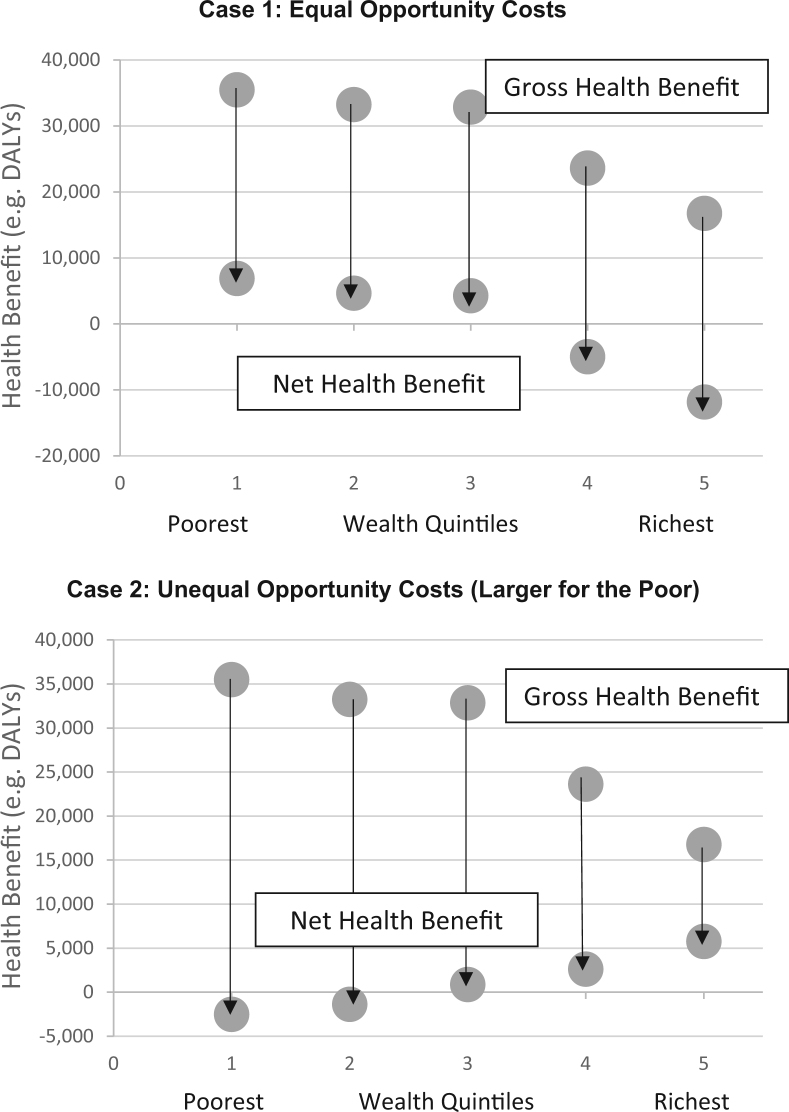
Health equity implications of opportunity costs:hidden traps.

The potential health equity implications of alternative sources of funding are illustrated in Figure 2. The “gross” health impacts consider only the distribution of program benefits, as if there were no health opportunity costs. These gross impacts are shown as the upper circles in the diagram, representing the sum total population health gains due to the program (e.g., in QALYs or DALYs). For example, if one were evaluating a breast cancer detection program, the gross impacts are the health gains due to the increase in cases of early detection of breast cancer and subsequent gains in length and quality of life for those patients that result from treatment at an early stage. The downward arrows then convert these gross health impacts into “net” health impacts, accounting for the distribution of health opportunity costs due to diverting resources from other uses. Case 1 illustrates a case in which the health opportunity costs are assumed to be equally distributed. Case 2 illustrates a case in which funding comes from a program that disproportionately benefits socially disadvantaged groups. Case 2 shows that programs that may initially seem to have a pro-poor health equity impact may in fact be equity neutral or even anti-poor when one accounts for the health effects of diverting money from alternative uses.

## Equity Impact Analysis

Cost-effectiveness analysis can be used to examine the distribution of benefits and opportunity costs from alternative policy options, broken down by one or more variables of concern to policymakers from an equity perspective. At the request of the UK health minister, for example, Holmes et al. [35] used CEA to model the social class distribution of the impacts of minimum alcohol pricing in the United Kingdom. They examined the effects on alcohol consumption, spending, and alcohol-related health harm and found that health benefits are substantially concentrated on heavy drinkers in routine and manual worker households.

### Extended Cost-Effectiveness Analysis

In low- and middle-income countries, a common approach to equity impact analysis is extended CEA (ECEA), developed by the *Disease Control Priorities*, third edition (DCP-3) project (http://www.dcp-3.org) [36]. ECEA analyzes the distribution of both health benefits and financial risk protection benefits (prevention of illness-related impoverishment) per dollar expenditure on specific policies in a given country. ECEA examines the financial risk protection benefits of policies given the high incidence of out-of-pocket payments in a large number of low- and middle-income countries and because the prevention of medical impoverishment is one major objective of health systems and universal health coverage [37].

As an illustration of ECEA, Verguet et al. [38] examined the distributional impact of a 50% cigarette price increase through excise tax in China over a 50-year period, compared to no change. They found that such excise tax increases could be pro-poor in China: the years of life gained would be more concentrated on the poor (79 million in the poorest quintile group) than on the rich (11 million in the richest quintile group), and the financial risk protection benefits would be largely concentrated among the poorest quintile group (accruing about 70% of the total $2 billion of insurance value gained).

ECEA has now been applied to the study of about 20 policy interventions in different low- and middle-income countries, producing breakdowns of costs, health benefits, and financial risk protection benefits by socioeconomic quintile group [39]. Hitherto, such analyses have tended to focus on distributions by socioeconomic group, but other equity-relevant variables can also be examined, including geographical location, ethnicity, sex, and severity of illness [40], [41].

### Distributional Cost-Effectiveness Analysis

Another framework for equity impact analysis is distributional CEA (DCEA), developed by the University of York. This approach focuses on the distribution of health effects and pays careful attention to the distribution of health opportunity costs from displaced expenditure within a fixed health care budget. Asaria et al. [14] used DCEA to examine the distributional health impacts, by social deprivation, ethnicity, and sex, of targeted versus universal reminder strategies for increasing uptake of bowel cancer screening. They found that the targeted strategy delivered a smaller gain in total health but a larger reduction in health inequality [23].

DCEA allows multiple inequality impacts on different social groups to be combined in the same analysis and compared in magnitude. It also aggregates all costs and effects into the common metric of net health benefits as well as presenting findings in a disaggregated form. This allows the construction of summary measures of net health equity impact that can be plotted on the horizontal axis of the equity impact plane, rather than reliance on judgments based on disaggregated data on the distribution of costs and effects. If required, this equity impact metric can then be weighed against the cost-effectiveness metric (net health benefit) on the vertical axis using an overall equity-weighted index of social welfare that combines concern for both equity and cost-effectiveness. As discussed later, we do not propose using an index of this kind as an all-purpose algorithm for decision making. Rather, we recommend sensitivity analysis using different equity weights to explore the implications of alternative views about the appropriate trade-offs between improving total health and reducing health inequity. A limitation of DCEA is that, like conventional CEA, it does not examine effects on financial risk protection. However, this limitation may be less important in countries like the United Kingdom where few people suffer impoverishment as a result of medical costs [42].

Equity impact analysis can also be conducted outside the context of CEA and the aforementioned ECEA and DCEA frameworks. Bajekal et al. [43] examined the distributional impact of changes in risk factors and treatment utilization on coronary heart disease mortality in different social groups in England from 2000 to 2007. This kind of study does not directly inform the priority-setting task of selecting among specific policies, however, because it does not provide information about the costs and effects of policies on changing risk factors or treatment utilization.

Another form of distributional impact analysis that is typically conducted outside the context of CEA is known as benefit-incidence analysis. This analysis looks at the benefits of public health care spending as a whole for different social groups. It generally examines only health care consumption and coverage rather than health outcomes, and looks at the average benefits of current expenditure rather than the marginal benefits of potential future changes in expenditure. Some recent benefit incidence analyses, however, have used data on subnational variation and change in expenditure and outcomes to estimate the health outcomes of marginal changes in spending in a way that could be used more directly to inform priority setting [44].

## Equity Trade-Off Analysis

Equity trade-off analysis examines trade-offs between improving total health and other equity objectives not usually addressed by CEA. The two main approaches to equity trade-off analysis—equity-constraint analysis and equity-weighting analysis—are described below.

### Equity Constraint Analysis—Counting the Cost of Equity

A simple approach to equity trade-off analysis is to count the cost of choosing fairer but less cost-effective options. This can be thought of as an equity constraint analysis because equity is treated as a constraint on the pursuit of health-maximizing cost-effectiveness. For example, Cleary et al. [45] explore the implications of imposing the equity constraint to treat either 100% of eligible patients or none. This can be seen as an equity constraint insofar as it avoids creating a “two-tier” public service that delivers effective treatment to some patients but not others.

Cleary et al. [45] explore the total health implications of imposing versus relaxing this equity constraint regarding indivisibility of health program delivery in relation to anti-retroviral therapy (ART) for human immunodeficiency virus (HIV). For example, in their Table VI, they find that with a budget of $6–$8 billion dollars, this equity constraint would drive the choice of no ART, even though less than 100% provision of first-line ART would be more cost-effective and deliver more total health. With a budget of $12 billion, indivisibility prevents the funds from being used to provide second-line ART to less than 100% of the eligible population, which would also deliver more total health.

The health loss associated with choosing the more “equitable” option gives an indication of the value the decision maker places on this equity constraint [11]. This approach can be implemented either by using a simple cost-effectiveness framework that compares two or more options given a fixed budget or by using more specialized mathematical programming techniques to handle complex choices involving different amounts of expenditure on different programs [10], [41], [46], [47].

### Equity-Weighting Analysis—Valuing the Importance of Equity

When programs fall in the “win-lose” or “lose-win” quadrants of the health equity impact plane, decision makers face difficult trade-offs between equity and cost-effectiveness. Equity-weighting analysis can be used to help policy-makers assess these trade-offs by quantifying how much concern for equity is required to choose cost-ineffective options that improve equity (programs in the “lose-win” quadrant) and cost-effective options that harm equity (programs in the “win-lose” quadrant). This can be done using “equity weights” for health benefits that apply to people with different characteristics or using an “equity parameter” that quantifies the degree of concern for reducing health inequity versus improving total health.

Different characteristics of health policies and the people affected by them can be used as the basis for setting equity weights, and several different equity-weighting systems have been proposed [48], [49]. Many of these systems do not pay special attention to the social characteristics of people but rather focus on their health characteristics—in particular, current severity of illness or overall lifetime experience of health, including past, present, and future health [50], [51], [52], [53], [54], [55]. These systems tend to be based on one or more equity parameters, such as an inequality aversion parameter within a social welfare function, which specifies how much one cares about reducing unfair health inequality but does not directly specify how much one cares about individuals with certain social characteristics [23], [56]. An equity parameter does, however, indirectly imply a specific set of equity weights for people with different characteristics when combined with information about the existing distribution of health among individuals with those characteristics. These implied weights will then change in response to changes in the distribution of health and social variables.

Equity-weighting analysis also requires that all costs and effects be aggregated into a common metric to which weights can be applied, such as annual mortality risk, life years, QALYs, or DALYs. In addition, if decision makers are interested in impacts on relative inequality (e.g., life expectancy ratios) as well as absolute inequality (e.g., life expectancy gaps), then an estimate of the baseline distribution of health will be required because relative inequality impacts depend on the baseline levels as well as the absolute changes.

## Discussion

In response to growing policy concerns about health equity, the tools of economic evaluation are being refashioned to provide useful evidence about health equity impacts and trade-offs. This article has introduced the key concepts that underpin the use of CEA to address health equity concerns. This article has also reviewed the main practical tools available for analyzing who gains and who loses from policy interventions (equity impact analysis) and for assessing equity trade-offs between improving total health and other health equity objectives not usually addressed by CEA (equity trade-off analysis). These approaches can also be thought of as a type of multicriteria decision analysis with two decision criteria—improving total health and improving health equity—and could be embedded within a wider multicriteria decision analysis that encompasses further decision criteria [57], [58].

The methods we describe can be used to address most but not all of the equity concerns that routinely arise in HTA, including 1) concern for reducing inequalities in health-related outcomes by income, ethnicity, geographical location, disability, sex, and other social variables; and 2) concern for prioritizing severely ill patients, including “end-of-life” patients. “Fair innings”–type concerns to reduce lifetime inequalities can be addressed under both headings 1) and 2) by using lifetime health metrics. These methods, however, do not address other issues sometimes discussed in relation to equity (e.g., orphan diseases, productivity costs, benefits to careers, and nondiscrimination) and have not yet been extended to analysis of inequality between current and future generations or the dynamics of social mobility [59].

Aligning the methods of CEA to address equity concerns is only one facet of the much larger question of how to design fair processes of decision making that appropriately address equity concerns [8], [18]. Equity-informative CEA can only address a subset of the diverse stakeholder concerns about fairness that may arise in relation to a specific decision, and decision makers will always need to consider wider issues and using further sources of information. One useful approach to ensuring that decision makers give due attention to wider equity concerns, for example, is the use of equity checklists [59], [60], [61].

More fundamentally, robust institutional structures, processes, and incentives are needed to ensure that decision makers take appropriate steps to reduce inequities in health. Analysis of the health equity implications of decisions cannot help to improve decision making if, for example, the analysis is poorly conducted or communicated, or based on the idiosyncratic value judgments of a narrow group of experts rather than the broader community of stakeholders, if policy advisers lack sufficient training to understand the findings, or if the conclusions are disregarded by decision makers who merely pay lip-service to health equity concerns.

Using CEA to analyze equity impacts and trade-offs requires the same basic analytical skills needed for standard CEA. It is more demanding, however, in terms of data requirements because it requires social distributions of key parameters rather than population average values. Data limitations can be particularly severe in low-income countries that lack basic health information systems, such as vital statistics on births and deaths and hospital and primary care administrative data. As the DCP3 project has amply demonstrated, however, equity-informative CEA can successfully be performed in low- and middle-income countries using existing survey-based data sets, such as the demographic and health surveys, which include information disaggregated by socioeconomic status and geographical setting.

An important frontier for future research is to provide better estimates of the distribution of opportunity costs. Work is ongoing to examine this distribution in England, drawing on existing research on variation in health expenditure and outcomes at a subnational level [62]. Linking this research with data on health care utilization by different social groups will provide empirical estimates of how the opportunity costs of health expenditure within a fixed health budget are distributed. This also opens the prospect of producing “equity league tables” by ICD-10 disease code to provide decision makers with a rough indication of the likely health equity impacts of programs in particular disease areas, without the need to undertake a full-blown equity impact analysis. This work is challenging, however, because many countries do not have suitable subnational data on variation in health expenditure and outcomes or may have health system characteristics that make it difficult to identify where the opportunity costs lie.

In the past, some authors have taken a skeptical view of the value of equity-weighting analysis due to the difficulty of securing consensus on an equity “algorithm” or “tariff” that rigidly prespecifies particular equity weights—for example, differential weights for individual health gains according to disease type (e.g., cancer), health characteristics (e.g., severity) or social characteristics (e.g., being poor) [63]. Equity-weighting analysis, however, does not have to be used as an algorithm for making decisions. Instead, we recommend that it be used as an aid to deliberation in the context of a specific decision via sensitivity analysis of different equity parameters to help decision makers and stakeholders explore the implications of alternative value judgements about equity. An advantage of this approach is that it helps to clarify the equity arguments advanced by different parties by laying bare their logical implications. The reporting of sensitivity analyses using different equity parameters may also help form useful “equity benchmarks” for decision makers to compare across different decisions.

Thus, we do see a role for equity-weighting sensitivity analysis as an aid to deliberation in the context of specific decisions in light of information on who gains and who loses. For example, the Dutch use sensitivity analysis around severity weighting in the economic evaluation evidence used to support decisions on inclusion in their basic health benefits package [64]. The UK National Institute for Health and Care Excellence uses sensitivity analysis on the narrower concept of “end-of-life” weights [65]. We also see a potential role for simple, pragmatic guidance on appropriate equity benchmarks that decision makers in particular jurisdictions may wish to recommend after an appropriate process of public consultation. For example, in 2014, the third Norwegian Committee on Priority Setting in the Health Sector proposed a way of distinguishing three categories of disease severity together with guidance on differential cost-effectiveness threshold ranges for these different categories [53].

Unlike the methods of severity weighting, the methods of ECEA and DCEA for analyzing who gains and who loses from decisions are not yet widely known among the HTA community and are not yet widely used to inform decision making. These methods are now starting to be applied, however, and are gradually becoming more sophisticated [66]. We therefore hope this article will help the HTA community to navigate the practical options open to them for providing policy makers with more useful information about the health equity implications of their decisions.
